# The Interplay between Chromatin and Transcription Factor Networks during B Cell Development: Who Pulls the Trigger First?

**DOI:** 10.3389/fimmu.2014.00156

**Published:** 2014-04-11

**Authors:** Mohamed Amin Choukrallah, Patrick Matthias

**Affiliations:** ^1^Friedrich Miescher Institute for Biomedical Research, Basel, Switzerland; ^2^Faculty of Sciences, University of Basel, Basel, Switzerland

**Keywords:** hematopoiesis, B cell development, transcription factors, chromatin regulators, pioneer transcription factors

## Abstract

All mature blood cells derive from hematopoietic stem cells through gradual restriction of their cell fate potential and acquisition of specialized functions. Lineage specification and cell commitment require the establishment of specific transcriptional programs involving the activation of lineage-specific genes and the repression of lineage-inappropriate genes. This process requires the concerted action of transcription factors (TFs) and epigenetic modifying enzymes. Within the hematopoietic system, B lymphopoiesis is one of the most-studied differentiation programs. Loss of function studies allowed the identification of many TFs and epigenetic modifiers required for B cell development. The usage of systematic analytical techniques such as transcriptome determination, genome-wide mapping of TF binding and epigenetic modifications, and mass spectrometry analyses, allowed to gain a systemic description of the intricate networks that guide B cell development. However, the precise mechanisms governing the interaction between TFs and chromatin are still unclear. Generally, chromatin structure can be remodeled by some TFs but in turn can also regulate (i.e., prevent or promote) the binding of other TFs. This conundrum leads to the crucial questions of who is on first, when, and how. We review here the current knowledge about TF networks and epigenetic regulation during hematopoiesis, with an emphasis on B cell development, and discuss in particular the current models about the interplay between chromatin and TFs.

## Introduction

The character of a cell type is defined by its specific transcriptional program, which is regulated by transcription factors (TFs) that bind DNA cis-regulatory elements (cis-REs) to activate or repress defined set of genes. Cis-REs refer to loci that regulate the expression of genes located in the same molecule of DNA. They are composed of binding sites for TFs that recognize a specific nucleotide sequence and therefore act as trans-acting factors. Promoters and enhancers are the two major types of cis-REs in eukaryotes. At the DNA sequence level, the repertoire of cis-REs is identical in all cell types. Therefore, the transcriptional programs specific to each cell lineage must be the consequence of the repertoire of TFs expressed in a given cell that select genes for transcriptional activation or repression. However, the same TFs can be equally expressed in different cell types but have distinct binding profiles, indicating that the interaction between the TFs and their cognate sequences is not sufficient to explain the action of TFs and their transcriptional output. Indeed, in addition to the DNA sequence recognition, TFs occupancy strongly depends on chromatin structure and epigenetic modifications which provide an additional layer of gene regulation and establish heritable cellular memories.

**Figure 1:**
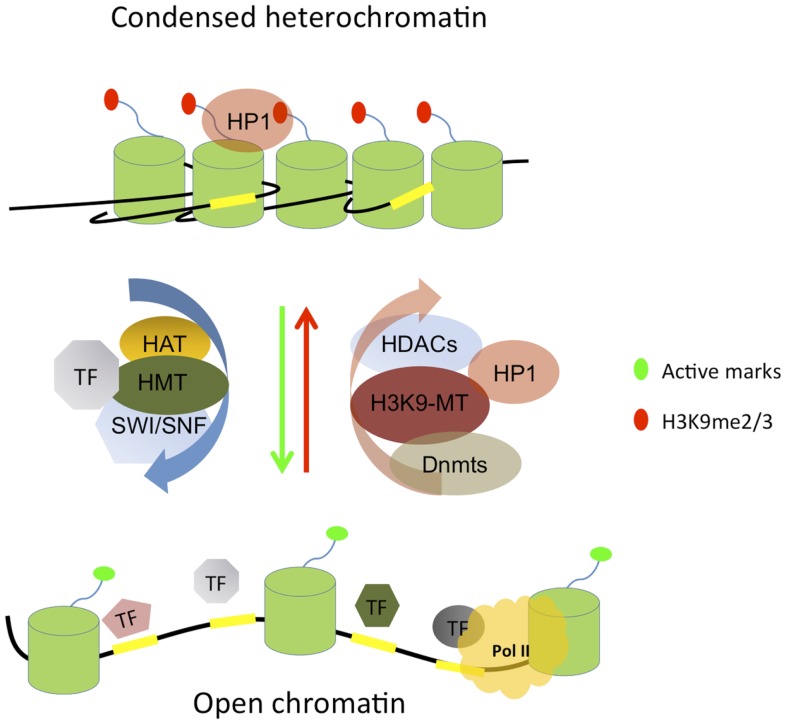
**Simplified scheme of condensed heterochromatin and open chromatin features**. Heterochromatin is mostly hypo-acetylated, marked by methylated lysine 9 on histone H3 (H3K9me2/3) which forms a binding site for heterochromatin protein 1 (HP1), contains methylated DNA, and results from the action of repressive complexes such as histone deacetylases (HDACs), H3K9 methyltransferases (H3K9-MT), and DNA methyltransferases (Dnmts). Chromatin opening is orchestrated by the concerted action of transcription factors (TFs) and chromatin modifying enzymes such as histone methyltransferases (HMTs), histone acetyl transferases (HATs), and SWI/SNF remodeling complexes. Open chromatin is generally acetylated, harbors active histone marks, and is accessible to the transcription machinery and RNA polymerase II (Pol II). TF binding sites (TFBSs) are indicated by yellow rectangles and the nucleosomes consisting of histone octamers are depicted by green cylinders. For simplicity, only a single histone tail is shown protruding out of each nucleosome and DNA methylation is not depicted.

Chromatin consists of repeating units of nucleosomes, comprising histone octamers (containing two copies each of H2A, H2B, H3, and H4) around which 147 bp of DNA are wrapped (1). Multiple residues within the tails and the globular domains of histones can undergo post-translational modifications (PTMs), including acetylation, methylation, phosphorylation, ubiquitination, and sumoylation. These PTMs are catalyzed by a variety of histone-modifying enzymes that have been classified in two major groups, the writers such as histone acetyl transferases (HATs) and histone methyltransferases (HMTs) and the erasers such as histone deacetylases (HDACs) and histone demethylases (KDMs) (2) (Figure 1). Histone modifications act combinatorially to regulate transcriptional activity; some histone modifications are associated with transcriptional activation while others are associated with distinct mechanisms of transcriptional repression. For example, tri-methylation on lysine 4 of histone H3 (H3K4me3) is mainly associated with active promoters; in contrast, the mono methylation of the same residue (H3K4me1) is a hallmark of poised and active enhancers, while H3K27ac marks exclusively active enhancers and promoters. The best studied repressive histone modifications are the methylation of lysines 9 and 27 of histone H3 (H3K9 and H3K27), which are respectively associated with heterochromatin and polycomb-group (PcG) proteins-mediated repression.

DNA methylation provides yet an additional mechanism for gene regulation. It is an efficient repressive DNA modification that occurs at the fifth position of cytosine (5-mC), mostly in the context of CpG dinucleotides (3) and is associated with transcriptional repression through two general mechanisms. First, DNA methylation can directly inhibit the binding of proteins important for transcription initiation, such as TFs and others. Moreover, methylated DNA can recruit proteins containing a methylated DBD, which may interfere with transcription by co-recruitment of repressors such as HDACs [reviewed in Ref. (4)]. Most of the genome is depleted of CpGs except for CpG islands, which represent ca. 60% of mammalian promoters and are largely unmethylated (5). DNA methylation is catalyzed by three enzymes: the maintenance DNA methyltransferase Dnmt1, which ensures that already methylated residues are faithfully maintained during DNA replication (6), and the *de novo* methyltransferases Dnmt3A and Dnmt3B which can add methyl groups to non-methylated CpG residues (7). DNA methylation is dynamic and also reversible: removal of methyl groups can occur through active or passive mechanisms. The latter is due to the absence of methylation by Dnmt1 of newly synthesized DNA during replication. In contrast, active DNA demethylation corresponds to the reaction that leads to the removal of the methyl group from 5-mC residues independent of DNA replication. Active DNA demethylation has been a controversial subject as many mechanisms were proposed to explain this process and the putative demethylases could not be identified in a conclusive manner [reviewed in Ref. (8)]. However, it is now well accepted that the dioxygenases Tet1 and Tet2 catalyze DNA demethylation through the conversion of 5-mC to hydroxymethyl cytosine (5-hmC) (9, 10).

Additional mechanisms involved in epigenetic regulation are contributed by chromatin remodeling complexes (CRC) and diverse kinds of non-coding RNAs. Chromatin remodelers are ATP-dependent complexes that regulate DNA accessibility by modifying nucleosome positioning and conformation. They can be divided into four groups: the SWI/SNF, ISWI, CHD, and INO80 families of remodelers [reviewed in Ref. (11, 12)]. In addition, long or short non-coding RNAs can influence chromatin and gene expression, for example by mediating inactivation of one chromosome (X inactivation by Xist RNA), opening up loci or helping to define boundaries of chromatin domains [reviewed by Mercer et al. (13)].

These different mechanisms of histone modifications, DNA methylation, chromatin remodeling, and non-coding RNAs play a central role in shaping chromatin structure, which in turn affects the interaction between TFs and their cognate binding sites. Conversely, the binding of TFs triggers a chain of events, often leading to changes in local chromatin properties. Indeed, TFs can interact with and recruit many chromatin modifying or remodeling complexes to their target loci. Thus, establishing chromatin structure requires TF activity and TF activity depends on chromatin structure. This reciprocal interplay raises a major question: how is the communication between TFs and chromatin regulated and which additional cellular signals feed into this complex network during development and cellular differentiation?

**Figure 2:**
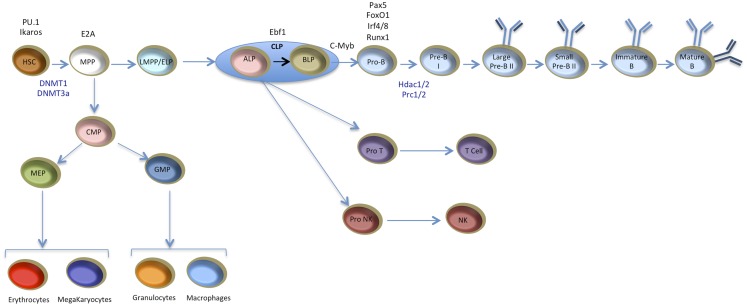
**Scheme for B cell development from HSCs to mature B cells**. Successive stages and alternative lineages are indicated. Key transcription factors and chromatin regulators are shown according to their established requirement during the early B cell differentiation process. HSC, hematopoietic stem cell; MPP, multipotent progenitors; MEP, megakaryocyte–erythrocyte progenitors; LMPP, lymphoid-primed multipotent progenitors; ELP, early lymphoid progenitors; CMP, common myeloid progenitors, GMP, granulocyte–macrophage progenitors; CLP, common lymphoid progenitors; ALP, all lymphoid progenitors; BLP, B cell-biased lymphoid progenitor; Pro NK, progenitor natural killer cells; NK, natural killer cells. When a factor is required at multiple developmental stages, only the earliest stage has been indicated and only factors important for the early stages of hematopoietic or B cell development are depicted. For simplicity, only one model of myeloid versus lymphoid divergence is illustrated; the alternative routes are not shown here [reviewed in Ref. (14)].

Understanding the mutual and interdependent interactions between TFs and chromatin features and their impact on gene regulation in a developmental system requires a biological paradigm where successive differentiation stages can easily be identified and isolated. In this regard, hematopoiesis provides a powerful system to study epigenetic and transcriptional dynamics. B cells derive from hematopoietic stem cells (HSCs) through a multistep differentiation program. HSCs have both self-renewal and multipotency capacities. The precise balance of these properties is essential to maintain the HSC pool size throughout animal life. HSCs initially give rise to multipotent progenitors (MPPs) that loose self-renewal capacity but keep the ability to generate early progenitors of lymphoid and myeloid lineages. Lymphoid lineage consists of B, T, and natural killer (NK) cells while myeloid lineage contains macrophages (M), granulocytes (G), erythrocytes (E), and megakaryocytes (Mk). The exact branching point between lymphoid and myeloid lineages as well as the differentiation potential of progenitor populations is still matter of some debate [reviewed in Ref. (14)]. The identification of common lymphoid progenitors (CLPs) (15) and common myeloid progenitors (CMPs) (16) supports the model that lymphoid and myeloid lineages follow distinct developmental paths from MPPs. This model was challenged by the identification of the lymphoid-primed multipotent progenitors (LMPPs) that loose MkE potential but keep lymphoid and GM potential (17, 18). Another study showed that the MPP compartment contains a subpopulation of cells with strong lymphoid potential and weak myeloid colony-forming activity (19). These cells, called early lymphoid progenitors (ELPs) start to express recombination-activating gene 1 (Rag1) and Rag2 and initiate the immunoglobulin heavy chain (IgH) rearrangement (19). ELPs are thought to precede the CLP stage. Recently, it was shown that the CLP compartment contains two distinct subpopulations: all lymphoid progenitors (ALPs) and B cell-biased lymphoid progenitors (BLPs) (20). ALPs retain full lymphoid potential, whereas BLPs behave essentially as B cell progenitors (20). Mature B cells derive from BLPs through sequential differentiation steps that can be defined by five major stages that are phenotypically and functionally distinct: pro-B, pre-BI, large and small pre-B II, and immature B cells (21) (Figure 2). Early B cell differentiation is intimately connected to the DNA rearrangement of Ig genes, the so-called V(D)J recombination, in order to generate functional Ig molecules. Pro-B cells, first express the pan-B cell marker B220 and this coincides with entry into the B cell lineage. Next, pre-BI cells express the CD19 gene and complete recombination of the IgH diversity (D_H_) to joining (J_H_) segments and the next stage sees the generation of IgH V(D)J alleles [reviewed in Ref. (21)]. This allows expression of the rearranged heavy chain which assembles with the surrogate light chain to form the pre-B cell receptor (pre-BCR), a crucial checkpoint in B cell development (22). If cells pass this functional test they can go on to the next developmental stage, small pre-BII cells, where the Ig light chain rearranges and allows for the formation and exposure at the cell surface of a functional Ig molecule, the BCR. Finally, immature cells can then leave the bone marrow (BM) and enter the periphery (22).

The generation of immature and mature B cells from early precursors is a progressive process, every step of which is characterized by a specific transcription program involving the activation, repression, or maintenance of distinct sets of gene expression patterns. This genetic regulation results from the concerted action of ubiquitous and lineage-specific TFs as well as epigenetic modifiers. Proper and timely recombination of the Ig loci is essential for normal progression through B cell development and is highly dependent on chromatin structure, DNA methylation, and also expression of various RNAs across the Ig locus. In particular, the accessibility model, first posited by Frederick Alt and colleagues, highlighted the importance of “sterile” transcripts which originate from unrearranged Ig gene segments and make their chromatin accessible to the recombination enzymes RAG1 and RAG2 [reviewed in Ref. (23)]. Thus, B cell development presents an extraordinarily complex and dynamic system to study the establishment and maintenance of transcriptional and epigenetic networks.

## Key Transcription Factors Essential for B Cell Development

Loss of function studies using mouse models have identified many TFs important for distinct stages during B cell development and a particular emphasis has been put on early B cell specification and commitment. Prominent among those are E2A, Ebf1, and Pax5, as well as other TFs acting downstream and upstream to these factors. Some of these TFs such as Ebf1 and Pax5 are restricted to the B cell lineage while others such as Ikaros, PU.1, E2A, and FoxO1 are also involved in other lineage fate determination.

**Figure 3:**
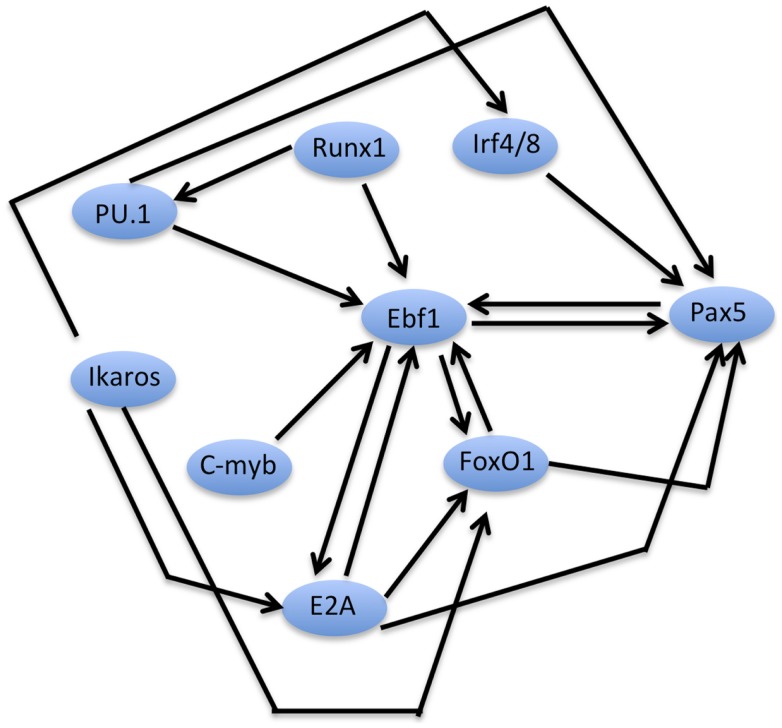
**An early B cell specification module**. Schematized network of inter-dependent TFs regulating early B cell development. The scheme depicts TFs that have been implicated in the control of early B cell specification and/or commitment (for clarity, factors such as Spi-B, OBF1, or NF-KB, which are important at later stages of B cell development, are not depicted here). Direct positive regulation between two factors at the transcriptional level is indicated by the corresponding arrows. Note that the scheme does not imply biochemical interactions (e.g., complex formation) between the factors, although they may also take place in some cases.

The expression of these TFs is temporally regulated; e.g., Ikaros, PU.1, and E2A are expressed in the very early progenitors including HSCs and MPPs before the commitment to the lymphoid branch, Ebf1 and FoxO1 are expressed at the CLP stage under the control of E2A (24) and Pax5 expression is induced by the concerted action of Ebf1, FoxO1, and E2A in committed pro-B cells. The sequential expression and activity of these TFs suggests a hierarchy in their action. Yet, the transcriptional regulation of early B cell development is not a simple hierarchical cascade, as many of these TFs act in a cooperative manner and directly regulate the expression of other TFs, involving both positive and negative feedback loops leading to a complex cross-regulatory network (25) (Figure 3).

### PU.1

If one considers a hierarchical classification of the TFs involved in B cell development, PU.1 and Ikaros come on the top of the regulatory pyramid. PU.1 (encoded by *Spi-1, Sfpi-1*) belongs to the ETS family of TFs, its expression was thought to be restricted to the hematopoietic lineage, but was recently also detected in adipocytes (26). Within the hematopoietic system, PU.1 activity is essential for the development of lymphoid cells as well as macrophages and neutrophils (27). Disruption of the PU.1 DBD in mouse prevents the commitment of MPPs toward the lymphoid lineage (27). PU.1 is expressed in HSCs (28), lymphoid, and myeloid progenitors (16) as well as in fully mature and functional cells (29). This broad expression pattern indicates that PU.1 is not only required for cell differentiation but also plays a role in the function of the specialized hematopoietic cells. The expression of PU.1 in many hematopoietic lineages raised the question about its mechanism of action and the rules that determine the interaction between this TF and its binding sites in different cellular and physiological contexts. Genome-wide mapping of PU.1 binding sites in macrophages and B cells revealed that PU.1 is enriched at transcription start sites (TSSs), but the majority of binding sites were found at inter- and intra-genic sites (30) indicating a role of PU.1 in regulating both transcription initiation and enhancer function. Interestingly, PU.1 binding at TSSs exhibits a high correlation between macrophages and B cells; in contrast, binding sites at distal regulatory elements are highly cell type-specific. Motif analysis of cell type-specific PU.1 binding sites revealed that PU.1 binds in vicinity of lineage-specific TFs: B cell-specific PU.1 binding sites are enriched in E2A, Ebf1, OCT, and NF-kB motifs, while macrophage-specific sites are enriched in C/EBP and AP-1 motifs (30). These findings strongly suggest that the cell type-specific function of PU.1 is partly due to its collaborative interaction with other lineage-specific TFs. The role of PU.1 in shaping the enhancer repertoire in hematopoietic cells will be further discussed in a later section of this review. Interestingly, PU.1 action was shown to depend critically on its expression level and involves a tight dose-dependent control. PU.1 shows low to medium expression level in LT-HSCs and exhibits varied levels in progenitors and mature cells; e.g., PU.1 is weakly expressed in erythroid and T cells and shows intermediate levels in B cells, in contrast, it is highly expressed in macrophages and neutrophils (31). Importantly, this graded expression has a critical role in specifying the different lineages: by artificial expression of PU.1 in PU.1-deficient progenitors, it could be demonstrated that moderate PU.1 levels promote B cell development, while high PU.1 expression promotes macrophage differentiation and at the same time blocks B cell development (32).

### Ikaros

The zinc finger factor Ikaros (encoded by the *izkf1* gene) also plays a critical role during early lymphoid lineage specification. Ikaros was proposed to promote the differentiation of pluripotent HSCs into the lymphocyte pathways: mutational disruption of the Ikaros DNA-binding domain (DBD) leads to an early block in lymphopoiesis before the commitment to the lymphoid restricted stages (33). However, another study showed that Ikaros is dispensable for the transition from HSCs to LMPPs, but is rather required for the progression of LMPPs into the lymphoid lineages (34). Recently, Ikaros was found to be required for the induction of lymphoid lineage priming in HSCs and for the repression of self-renewal and multipotency genes after HSC differentiation (35). Ikaros is also involved in later stages of B cell development, where it promotes heavy-chain gene rearrangements by inducing expression of the RAG1 and RAG2 genes, as well as by controlling accessibility of the variable gene segments and compaction of the IgH locus (36). Furthermore, Ikaros was recently shown to be required for the differentiation of large pre-B to small pre-B cells and for transcription and rearrangement of the IgL locus (37). Ikaros functions either as a transcriptional activator or repressor by recruiting various CRC including SWI/SNF and Mi-2/nucleosome remodeling and deacetylase (Mi-2/NuRD) to DNA regulatory elements and to pericentromeric heterochromatin (38–42).

### E2A

E2A (encoded by *tcf3* with two splice variants, E12 and E47) is a helix–loop–helix TF essential for B cell differentiation (43, 44). E2A-null mutant mice fail to generate LMPPs and lack B cells (43). E2A acts synergistically with PU.1 and is required for Ebf1 and FoxO1 expression at the CLP stage (45, 46). Genome-wide mapping experiments in B cell progenitors (Ebf1^−/−^ and Rag2^−/−^) showed that E2A binds both TSSs and putative enhancers (24) and is required to induce H3K4me1 deposition at enhancer elements in concert with PU.1 (30).

### Early B cell factor 1

Early B cell factor 1 (Ebf1) belongs to the EBF/COE family of TFs (47). EBF/COE family members contain an N-terminal DBD with an atypical zinc knuckle domain (H–X3–C–X2–C–X5–C), a TF immunoglobulin (TIG/IPT) domain, a helix–loop–helix–loop–helix (HLHLH) domain and a carboxy-terminal transactivation domain (48). The HLHLH domain was found to be important for the dimerization of EBF1 (48). EBF1 is essential for B cell specification (49) and commitment (50). Ebf1 acts in concert with E2A, FoxO1, and other TFs to regulate the expression of many genes required for B cell development including TFs such FoxO1 and Pax5 (51); the latter in turn binds to *Ebf1* enhancers and increases its expression, thereby leading to a positive feedback loop between these two factors (24, 52, 53). Ebf1 can also act as a repressor; indeed, it was shown that Ebf1 prevents Id2- and Id3-mediated inhibition of the E47 isoform of E2A by downregulating the expression of their mRNA (54).

### Pax5

Pax5 acts downstream of Ebf1, its expression is under the control of a cohort of TFs including PU.1, Ebf1, FoxO1, IRF4, and IRF8 (55). Pax5 is essential for B cell commitment (56) and maintenance of B cell identity through activation of B cell-specific genes and repression of lineage-inappropriate genes (57). Deletion of Pax5 in mature B cells leads to the de-differentiation to lymphoid progenitors, which can differentiate into functional T cells (58). The role of Pax5 in regulating gene expression will be discussed in more detail in a later section.

### FoxO1

The forkhead TF FoxO1 plays an important role during B cell development. FoxO1 was found to be critical at several stages of B cell differentiation (59). Early deletion of FoxO1 causes a substantial block at the pro-B cell stage due to a failure to express the IL-7 receptor-alpha chain. FoxO1 inactivation in late pro-B cells results in an arrest at the pre-B cell stage due to impaired expression of Rag1 and Rag2 (59), which are direct targets of FoxO1 (60). In addition, deletion of FoxO1 in peripheral B cells leads to reduced number of lymph node B cells due to down regulation of L-selectin and defect in class-switch recombination (59).

### c-Myb and Runx1

B cell development also depends on many other TFs such as for example c-Myb and Runx1. Deletion of c-Myb in mice leads to a block at the pre–pro-B cell stage which is accompanied with impaired expression of the alpha-chain of the IL-7 receptor and Ebf1 (61). Deletion of Runx1 also causes a developmental block at the pro-B cell stage accompanied by reduced expression of E2A, Ebf1, and Pax5. Furthermore, Runx1 directly binds the *Ebf1* promoter and this binding is critical for *Ebf1* activation; indeed, Runx1-deficient pro-B cells were shown to harbor excessive amounts of the repressive histone mark H3K27me3 in the *Ebf1* proximal promoter. Interestingly, retroviral transduction of Ebf1, but not Pax5, into Runx1-deficient progenitors restores B cell development (62). It was also shown that Runx1 controls the expression of PU.1 via direct interaction with its upstream regulatory element (URE) (63).

As discussed above, many of the TFs critical for early B cell development directly regulate each other’s expression, positively or negatively, by binding to cis-REs in their corresponding genes. This inter-dependent network forms a B specification module, which has EBF1 at its center, which in concert with Ikaros, E2A, IRF4/8, and FoxO1, positively activates expression of Pax5, thus locking B cell development (Figure 3).

## Epigenetic Regulators Involved in Hematopoiesis and B Cell Development

In addition to the TFs, many epigenetic regulators are crucial for hematopoiesis and/or B cell development. Among those, PcG proteins play an important role in this system. Mammalian cells contain two major PcG complexes, PRC1 and PRC2. PRC2 contains SUZ12, EED, and EZH1 or EZH2. EZH proteins are HMTs that catalyze the di- and tri-methylation of histone H3K27 (64). PRC1 contains RING1, CBX, PHC, and BMI1 or MEL18 [reviewed in Ref. (65)]. PRC1 recognizes and binds H3K27me3 via its subunit CBX, while RING1 mono-ubiquitylates histone H2A at lysine 119 (H2AK119ub1) (61, 62). The H2AK119ub1 mark is thought to play a role in inhibiting RNA polymerase II (pol II) elongation (66). The H3K27me3 mark is associated with the silencing of many key developmental regulatory genes, such as Hox homeotic genes and many others [reviewed in Ref. (67)].

Many PcG deficiencies correlate with defective development and/or activation of lymphocytes. For example, inactivation of Bmi1 or mel-18 causes a severe block in B cell development that leads to B cell lymphopenia (68, 69). By contrast, deficiency in Cbx2 does not affect lymphocyte development but alters splenic B cell response to lipopolysaccharide (LPS) (70). Conditional knockout studies targeting members of the polycomb machinery highlighted the critical role of these enzymatic complexes in the hematopoietic system. Bmi1 is the most studied PRC1 subunit in hematopoiesis. Depletion of Bmi1 leads to impaired self-renewal capacity of HSCs due to the de-repression of two major cell cycle regulators: Ink4a (p16) and Arf (p19) (71). Bmi1 directly binds and repress the promoters of these genes and the deletion of both *Ink4a* and *Arf* genes restores the self-renewal capacity of *Bmi1*^−/−^ HSCs (72). Moreover, *Bmi1*^−/−^ mice have a BM microenvironment that is severely defective in supporting hematopoiesis. In this case however, the deletion of both *Ink4a* and *Arf* genes did not significantly restore the impaired BM microenvironment (72). Bmi1 is also involved in the repression of Ebf1 and Pax5 in HSCs and MPPs. Depletion of Bmi1 causes aberrant expression of these two genes, leading to premature lymphoid lineage specification (73). Another PRC1 subunit, Ring1b, was also found to be critical for adult hematopoiesis. Mice deficient for Ring1b in hematopoietic cells develop a hypocellular BM that unexpectedly contains an enlarged, hyperproliferating compartment of immature cells, with an intact differentiation potential. These defects are associated with differential upregulation of cyclin D2 and Ink4a (74). Controlled expression of PRC2 components is also important for hematopoiesis. Several studies have highlighted the role of Ezh1 and Ezh2 in embryonic and adult HSCs. Loss of Ezh2 severely impairs fetal HSC self-renewal without affecting the function of adult stem cells present in the BM (75). In addition, EZH2 was also found to have a crucial role in early B cell development and in rearrangement of the IgH gene (66).

Early B cell development also requires HDACs activity (76). Targeted deletion of the major class I HDACs, HDAC1 and 2 showed that B cell development requires the presence of at least one of these two enzymes. When both enzymes are deleted, B cell development is dramatically impaired at the large pre-BII stage with a strong cell cycle block in the G1 phase accompanied by the induction of apoptosis. In contrast, elimination of HDAC1 and HDAC2 in mature resting B cells is not deleterious; however, when these cells are induced to proliferate cell cycle block and apoptosis ensue. These data indicate that the role of HDAC1 and 2 during early B cell development is at least partially linked to cell cycle control (76). The potential role of HDACs in controlling other processes in B cells and other hematopoietic lineages remains to be elucidated.

The activity of DNA methyltransferases is also crucial for hematopoiesis. Conditional deletion of the maintenance DNA methyltransferase Dnmt1 in HSCs leads to impaired self-renewal capacity and prevents HSCs from giving rise to hematopoietic progenitors (77). Based on the initial studies, loss of the *de novo* DNA methyltransferases, Dnmt3a or Dnmt3b alone was thought to have no impact on HSC function (78); by contrast, loss of both together was reported to abolish self-renewal without affecting differentiation capacity (78). However, a more recent study reported that Dnmt3a-null HSCs exhibit upregulation of multipotency genes and downregulation of differentiation factors. The progeny of Dnmt3a-deficient HSCs exhibit global hypomethylation and impaired repression of HSC-specific genes. These data highlighted the important role of Dnmt3a in the repression of HSC genes in order to enable proper cell differentiation (79).

V(D)J recombination of immunoglobulin genes is thought to be regulated by changes in the accessibility of target sites, such as modulation of methylation. *In vitro* experiments showed that specific methylation within the heptamer of recombination signal sequences markedly reduces V(D)J cleavage without inhibiting RAG1/RAG2–DNA complex formation (80). Recent investigations of the IgH locus recombination showed that the diversity (D_H_) and joining (J_H_) gene segments are methylated prior to recombination, in contrast the DJ_H_ product is demethylated. DJ_H_ junctional demethylation is restricted to B cells and requires the Eμ enhancer, located within the intronic region of the IgH locus (81). However, it is unclear whether the demethylation is required for DJ_H_ junction or whether it is simply the consequence of the DNA recombination. Earlier experiments had shown that loss of methylation of the kappa light chain locus is not sufficient to activate recombination in cultured pre B cells lacking Dnmt1 (82). Cd19-cre mediated deletion of the *de novo* DNA methyltransferase Dnmt3a and Dnmt3b, failed to identify a critical role for these enzymes in B cell development (83). Cd19-cre is expected to induce the deletion of targeted genes from pre B cells onward (84). Thus, this study, strongly suggest that Dnmt3a and b are dispensable to the progression from pre B cells to mature B cells. Overall, these studies suggest that the maintenance DNA methyltransferase Dnmt1 is required at all the stages of hematopoiesis, whereas the *de novo* DNA methyltransferases Dnmt3a and 3b are required only at the very early stages and become dispensable at later stages. However, additional studies will be needed to fully test these assumptions.

## Interplay between Chromatin Landscape and TF Activity during B Cell Development

**Figure 4:**
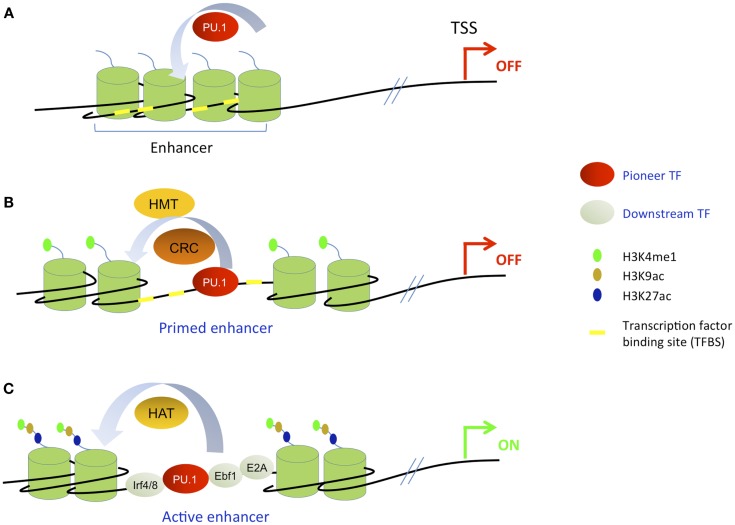
**Simplified scheme of stepwise enhancer activation based on the pioneering model**. A pioneer TF is exemplified by the ETS factor PU.1; the transcription start site (TSS) is indicated by a red or green arrow and the enhancer element is schematized by four nucleosomes. For simplicity, the nucleosomes covering the rest of the DNA including the promoter region are not indicated. **(A)** The pioneer TF recognizes and binds its cognate site in condensed chromatin. **(B)** Recruitment of histone methyltransferases (HMTs) and chromatin remodeling complexes (CRC) which prime the enhancer for subsequent activation. At this step, the enhancer now harbors H3K4me1 mark but still lacks acetylation marks. **(C)** Subsequent collaborative binding of downstream TFs accompanied by histone acetyl transferases (HATs) that catalyze histone acetylation, leading to enhancer activation and gene transcription.

The progression of MPPs toward specialized cells is thought to be accompanied by extensive epigenetic reprograming. In the recent years, genome-wide technologies have been used to map histone modifications and TF binding sites (TFBSs) in various B cell populations and to describe the epigenetic changes accompanying B cell development. Recent studies in different systems indicated that the chromatin of cis-REs is in a pre-active state in stem cells and/or early progenitors before the transcriptional initiation, leading to the concept of “gene priming” (85). The priming is thought to be driven by a specific class of TFs called “pioneer TFs,” that are able to induce the early chromatin changes during the gene activation process (86). Pioneer TFs are thought to mark certain loci for downstream activation during development. Cis-RE bookmarking by pioneer TFs was first described in the mouse liver where FoxA and Gata factors were found to bind the liver-specific enhancer of the *alb1* gene in the precursor gut endoderm prior to its activation in nascent liver (87, 88). The appellation “pioneer TF” must meet the following criteria, although these have not always been unambiguously demonstrated in every case: (i) binding to the regulatory region prior to transcription activation, (ii) binding prior to the arrival of other factors, (iii) binding to their target sites in condensed chromatin, and (iv) being able to induce chromatin modifications and/or remodeling in order to render the locus accessible for downstream TFs [reviewed in Ref. (86)]. It is important to mention that in the majority of cases, it is difficult to establish unequivocally the exact binding chronology of a set of TFs at a given locus (see Figure 4).

**Figure 5:**
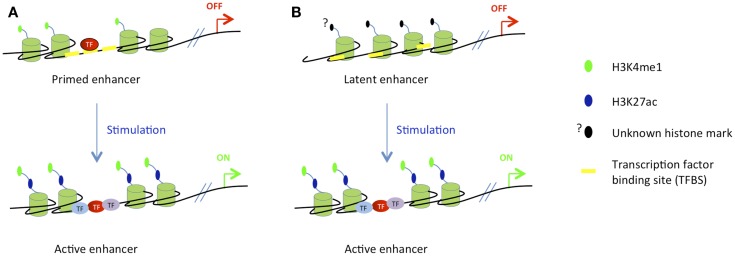
**Stimuli-dependent activation of different classes of enhancers in terminally differentiated cells**. **(A)** In differentiated cells, the majority of stimuli-dependent enhancers are primed, i.e., already bound by pioneer TFs and marked by H3K4me1, but inactive. After stimulation, downstream inducible TFs bind to their cognate sites to activate target enhancers and gene transcription. **(B)** Latent enhancers are special class of enhancers, that are unbound by pioneer TFs and unmarked by H3K4me1 in unstimulated cells. At this stage, their chromatin structure is not well characterized. TF binding, active chromatin marking, and gene activation occur after the stimulation.

Several studies have described primed enhancers (sometimes also called poised enhancers) in the hematopoietic system (85, 89). Primed enhancers refer to distal regulatory elements that harbor H3K4me1 mark but lack acetylation marks such as H3K27ac and H3K9ac; their associated genes are therefore not transcribed. In contrast, active enhancers harbor both H3K4me1 mark and acetylation marks and their associated genes are transcribed. According to the current priming models, once a cell has reached terminal differentiation, its enhancer repertoire is completely established and maintained by cooperatively acting lineage-specific TFs. Inducible or regulated TFs that are activated by extracellular stimuli operate within this predetermined framework, landing close to where master regulators are already bound (Figure 5). However, this model was recently challenged by the identification of a novel class of enhancers in macrophages. These cis-REs have been called “latent enhancers” and are not bound by TFs and also lack H3K4me1 and acetylation marks under basal or uninduced conditions. However, they acquire all these features in response to stimulation (90) (see Figure 5). These data suggest that the priming may not be absolutely required for all enhancer elements; however, it cannot be excluded that upon the stimulation the priming occurs before the activation of target enhancers.

## Understanding the Order of Events

In an effort to investigate how the enhancer repertoire is established and maintained during myeloid and B cell development, Mercer et al. have generated long-term cultures of hematopoietic progenitors by enforcing the expression of the E-protein antagonist Id2, which inhibits E2A activity by preventing its binding to DNA. These progenitors, called Id2-HPC, can be differentiated *in vitro* into myeloid and B cell lineages by switching off Id2 expression, therefore effectively restoring E2A activity. Using this system, H3K4me1 mark was mapped in Id2-HPC cells as well as in myeloid and B cells generated *in vitro* from these artificial precursors. Interestingly, it was found that a substantial fraction of the lymphoid and myeloid enhancers were pre-marked by H3K4me1 (i.e., primed) already in MPPs. Thus, multilineage priming of enhancer elements in hematopoietic progenitors precedes commitment to the lymphoid and myeloid cell lineages (85). Motif analysis showed that PU.1 and Runx motifs were over-represented in H3K4me1 enriched loci in Id2-HPC cells, while enhancers of genes activated after B cell differentiation were enriched in E2A and Ebf1 motifs, in addition to PU.1 motif (85). These finding clearly demonstrate a relationship between cell type-specific binding of TFs and the pattern of H3K4me1 enhancer mark. They also indicate a potential role of PU.1 and Runx factors in priming enhancers in hematopoietic progenitors for subsequent downstream activation.

The correlation between PU.1 binding and presence of H3K4me1 mark was also reported in other hematopoietic lineages, including B cells (30) and macrophages (89). However, it was unclear whether the H3K4me1 modification serves as a beacon to recruit PU.1 and other TFs, or whether these TFs can initiate the deposition of H3K4me1 in hematopoietic progenitors. By expressing a tamoxifen-inducible PU.1/ER fusion protein in PU.1-deficient myeloid progenitors (91), Heinz et al. demonstrated that PU.1 binding can induce H3K4me1 deposition at some loci; yet, many loci were found to lack H3K4me1 despite the binding of PU.1, suggesting that additional factors may be required to write this mark (30). In addition, PU.1 was found to bind to loci that were already marked by H3K4me1; in this case PU.1 was found to be able to initiate nucleosome remodeling (30).

An earlier study has shown that the intronic enhancer of the *Pax5* gene is bound and regulated by PU.1, IRF4, IRF8, and NF-KB (55). Interestingly, the chromatin at this enhancer harbors active marks already in progenitors and is bound by PU.1 and IRF factors before *Pax5* transcription takes place in committed pro-B cells (55). It was also shown that the concerted action of PU.1 and Runx1 primes the activation of both promoter and enhancer elements of the *c-fms* gene in myeloid cell (92, 93). All together, these data clearly indicate the pioneering and priming abilities of the master hematopoietic regulator PU.1. This is consistent with its expression during early hematopoietic cell differentiation from HSCs onward and its dynamic collaborative binding with various TFs.

E2A was also found to alter the H3K4me1 pattern at enhancer elements in B cell progenitors, however it is unclear whether E2A can directly induce *de novo* H3K4 mono-methylation or only modulate the positioning of nucleosomes already pre-marked by H3K4me1 via nucleosome remodeling mechanisms (24).

Other downstream TFs such as Ebf1 and Pax5 were also found to regulate chromatin structure at cis-REs. For example, Ebf1 plays a role in the demethylation of the *Cd79a* promoter in B cell progenitors (94). Ebf1 is also crucially required for the remodeling and activation of chromatin in the *Pax5* promoter region (55). Pax5 regulates chromatin structure by recruiting chromatin-modifying and remodeling complexes to the Pax5 regulated loci (57). Interestingly, Pax5 fulfills both activation and repression functions; it induces active chromatin at promoters and enhancers of activated target genes, while eliminating active chromatin at the regulatory elements of repressed target genes. Pax5 rapidly induces H3K4 methylation and H3K9 acetylation at enhancers and promoters of activated target genes. Pax5 activation function involves direct interaction with the chromatin-remodeling SWI/SNF-like BAF complex, the histone acetyltransferase CBP, and the PTIP protein, which is known to recruit the MLL-containing H3K4 methyltransferase complex to chromatin (95). The repressing activity of Pax5 is mediated by its ability to recruit the NCoR1 co-repressor complex with its associated HDAC3 enzyme, which is likely responsible for histone deacetylation at some Pax5 repressed loci (57). Pax5 was also found to interact with members of the co-repressor Groucho family, thus leading to repression of target genes (96). An intriguing question is how TFs such as Ikaros and Pax5, having both activation and repression abilities, can distinguish which set of genes must be repressed or activated.

FoxO TFs were also described to have pioneering capacity [reviewed in Ref. (97)] and FoxO1 was found to be able to bind to its cognate sites in condensed chromatin. This binding stably perturbs core histone by de-condensing linker histone-compacted chromatin (98), possibly because the FoxO DBD shares structural similarities with the globular domain of the linker histones H1 and H5 (99, 100). Furthermore, the amino-terminal and carboxy-terminal regions of FoxO1 mediate histone H3 and H4 binding (98). By functioning as pioneer factors, FoxO TFs might open condensed regions and allow the binding of other TFs.

Overall, these studies demonstrated that chromatin structure in hematopoietic progenitors and committed cells can act as a beacon for binding of some TFs. Conversely, TFs such as PU.1, E2A, Ebf1, and many others, can modulate chromatin features at cis-REs to create or enhance a chromatin environment favorable for the binding of additional TFs. The priming and activation of cis-REs requires the collaborative and/or cooperative action of several TFs. For example, in B cells, the pioneer TF PU.1 co-occupies enhancers with E2A, Ebf1, and Oct2, while in macrophages it binds together with AP-1 and C/EBP (30). However, the synergy between pioneer and downstream TFs is not simply hierarchical but also involves cross-regulatory interactions. For example, at certain loci, PU.1 binding in B cells depends on E2A and Ebf1 (30) in spite of the fact that these two factors were not clearly identified as pioneer TFs. What regulates whether PU.1 binds by itself or requires other factors is not known, but is likely to involve the precise binding site and/or the local chromatin structure. It is also not established whether the pioneer TFs identified so far are a special class of factors with unique properties, or whether most factors can act as pioneers in the right context. Thus, the term “pioneer TF” does not have an absolute meaning, but should rather be viewed as a useful descriptor for properties identified in specific cases. Indeed, a downstream TF can act as a pioneer for an upstream TF, and vice versa, in a context- and locus-dependent manner. Therefore, many TFs involved in the priming of cis-RE can fall into the category of pioneer TFs. However, as mentioned above, it is often difficult to unambiguously monitor the precise chronological binding order of a set of TFs and corresponding epigenetic modifications under *in vivo* conditions. Thus, instead of using the term pioneer when the evidence is scarce, it may be better to rather speak about collaborative action of TFs at a given locus.

## Concluding Remarks

In summary, the questions of who is on first, the chromatin or the TF, when, and why/how are still largely unanswered. In some physiological situations, specific chromatin features must precede and are required for TF binding, while in other situations the TF binding initiates a series of epigenetic events eventually required for the recruitment of downstream TFs. The extensive efforts that were made to investigate transcriptional and epigenetic regulation of B cells and other hematopoietic lineages identified several mechanisms of cross-regulation between TFs, chromatin modifiers, and the pre-existing chromatin landscape. The interactions between the actors cited above are very likely to be controlled by environmental, spatial, and temporal signals that remain to be defined. Also, many additional factors – TFs, chromatin modifiers, non-coding RNAs … – remain to be tested for their potential role in the hematopoietic system or in B cells. However, achieving a deeper understanding of the mechanisms involved will require the ability to examine single cells in real-time to understand how the interplay between chromatin and TFs is orchestrated and unambiguously determine causal relationships. Also, the ability to genetically manipulate the system, not only at the level of the TFs or other trans-acting factors, but also of the cis-REs, e.g., by using the newly developed CRISPR/Cas9 (clustered regularly interspaced short palindromic repeats) system (101) will be invaluable to further our understanding.

## Conflict of Interest Statement

The authors declare that the research was conducted in the absence of any commercial or financial relationships that could be construed as a potential conflict of interest.
